# Correlates of active commuting to school across two generations: the Cardiovascular Risk in Young Finns Study

**DOI:** 10.1177/14034948241304246

**Published:** 2024-12-10

**Authors:** TH Suominen, T Kukko, X Yang, K Pahkala, S Rovio, M Hirvensalo, M Kähönen, O Raitakari, TH Tammelin, K Salin

**Affiliations:** 1 Faculty of Sport and Health Sciences, University of Jyväskylä, Jyväskylä, Finland; 2 Likes, School of Health and Social Studies, Jamk University of Applied Sciences, Jyväskylä, Finland; 3 Research Centre of Applied and Preventive Cardiovascular Medicine, University of Turku, Turku, Finland; 4 Centre for Population Health Research, University of Turku and Turku University Hospital, Turku, Finland; 5 Paavo Nurmi Centre and Unit for Health and Physical Activity, University of Turku, Turku, Finland; 6 Department of Public Health, University of Turku and Turku University Hospital, Turku, Finland; 7 Department of Clinical Physiology, Faculty of Medicine and Health Technology, Tampere University, Tampere, Finland; 8 Department of Clinical Physiology, Tampere University Hospital, Tampere, Finland; 9 Department of Clinical Physiology and Nuclear Medicine, Turku University Hospital, Turku, Finland

**Keywords:** Active transport, physical activity, trends, walking, cycling, active travel, children, adolescents, youth

## Abstract

**Aims:**

Active commuting to school (ACS), a source of physical activity (PA), has declined in many countries over recent decades. This study investigates ACS and the factors associated with it among Finnish children and adolescents across two generations: those born between 1965–74 and 1998–2010. We also explore potential generational differences in these associations.

**Methods:**

School commuting was self-reported by 2075 participants of the ongoing population-based Young Finns Study in 1983 (generation 1 (G1), aged 9–18, 52% female), and by their 1137 offspring in 2018 (generation 2 (G2), aged 8–20, 53% female). Factors associated with ACS and the moderating effect of generation on these associations were examined using generalized estimating equation models for clustered binary data, for summer and winter seasons separately.

**Results:**

A greater distance to school (*p* < 0.001) and belonging to G2 (*p* ⩽ 0.049) were negatively associated with ACS during both seasons. High parental leisure-time PA (*p* ⩽ 0.025 for both seasons) and urban living area (*p* < 0.001 for summer) were positively associated with ACS. Generation moderated the associations of school grade and parental income with ACS in the summer (*p* ⩽ 0.015). Among G1 only, attending lower secondary school (vs. primary school) was negatively associated with ACS, while higher parental income was positively associated with ACS. Neither gender nor parental education was associated with ACS.

**Conclusions:**

ACS was less common among the younger generation. Several correlates of ACS were identified, with generational differences. These findings can inform further research and guide policy decisions to promote ACS and ultimately enhance the PA of children and adolescents.

## Introduction

Active commuting to school (ACS), such as walking or cycling, has emerged as one of the key solutions to the global issue of insufficient physical activity (PA) among youth [1,2]. It offers a practical and sustainable way to incorporate PA into daily life, increasing PA even among those less inclined toward sports or other types of leisure-time PA [3,4]. As a low-cost and relatively stable source of PA [5], active commuting can help achieve health-enhancing levels of PA from childhood through adulthood.

ACS has been linked to numerous benefits, including better physical fitness [6] and cardiometabolic health [7], environmental advantages such as reduced CO_2_ emissions [8], and the development of road safety skills and independence [9]. However, ACS has declined in recent decades across various countries [4,10], with the extent of this decline and the overall prevalence of ACS varying by country and culture [4,11]. In several Nordic countries, including Finland, ACS levels have been high, most likely due to the cultural emphasis on ACS, safe urban planning, and a relatively well-established infrastructure for walking or cycling. Over 80% of Finnish students aged 10–16 living within 5 km of school commute actively [12], but a decreasing trend has been observed in recent decades [13,14].

ACS is influenced by various factors, such as sociodemographics and environmental, social, and policy aspects, which may vary across countries and cultures [4,15]. One of the strongest and most consistent determinants of ACS is the distance from home to school [4,16], with shorter distances increasing the likelihood of ACS. Distance is also closely related to other correlates of ACS, such as age. The association between age and ACS is presumed to follow an inverted U-shape, with the age-related increase driven by increased independent mobility and parental permission [15,17]. A decline is typically observed from around the age of 12 onwards [18], primarily due to distances to secondary schools typically being longer [15,17].

The correlates of ACS are dynamic, with the magnitude and direction of their effects on ACS being likely to vary over time [19]. Examining these correlates at different times could help explain ACS prevalence trends over time and contribute to increasing ACS participation. However, to our knowledge, no such studies have been conducted in Finland. To fill this gap, this study utilizes unique two-generation data from similar-aged participants within the same families, 35 years apart. Our objectives were 1) to explore the correlates of ACS among Finnish children and adolescents born between 1965–74 and 1998–2010, and 2) to investigate whether generation moderates the effects of these correlates on ACS. Owing to the considerable seasonal variation in ACS, typical for regions with significant seasonal climatic variations [12,20], and potential seasonal differences in ACS correlates, separate analyses were conducted for summer and winter commuting. Special attention was paid to the potentially dominating effect of the home–school distance, enabling well-adjusted estimates of the associations of other correlates of ACS.

## Methods

### Study design and participants

We utilized data from the ongoing, prospective Cardiovascular Risk in Young Finns Study (YFS) [21]. In 1980, baseline measurements were taken from 3596 children and adolescents (83.2% of those invited, 51% females), aged 3, 6, 9, 12, 15, and 18, randomly selected from the catchment areas of five Finnish university hospitals. The cohort has been regularly followed up in 3- to 9-year intervals since 1980. In 2018, the children of the original YFS participants were also invited to participate, with 2762 offspring aged 3–37 (48.5% of those invited) attending the study visit. The study was approved by the ethical committees of the five universities, and written informed consent was obtained from all participants, in accordance with the Helsinki Declaration.

This analysis used data from two cross-sectional samples: one collected in 1983 (the original YFS participants, generation 1 (G1)), and the other in 2018 (children of the original YFS participants, generation 2 (G2)). We used data from the first follow-up study in 1983 instead of the baseline assessments, as seasonal variation in commuting to school was assessed from that follow-up onwards. The G1 sample included data from all original YFS participants aged 9–18 at the time who provided information on their commuting to school (*n* = 2075, 52% female). Children aged 6 and 21 were excluded as they were not in compulsory education in Finland and thus not commuting to school. The G2 sample included data from 1137 participants aged 8–20 (53% female). Among 18–20-year-olds, only those who were still participating in schoolwork (e.g., vocational education or upper secondary school) were included. Questionnaire data were collected through a child survey, which could be completed at home with parental assistance.

## Measurements

### Commuting to school

The mode of commuting to school was assessed by a questionnaire, for summer and winter conditions separately. Similar questions were applied for both generations. The commuting mode was coded as 1 = own car or carpool, 2 = public transport, 3 = walking, 4 = cycling, and the modes further categorized as “passive” (car or public transport) or “active” (walking or cycling), for both seasons separately.

In a sample of 60 participants, the 2-week test–retest reliability of the commuting mode question from the YFS Physical Activity Questionnaire was high, with intraclass correlation coefficients of 0.83 in winter and 0.98 in summer [18]. In addition, significant correlations were found between the commuting question in both seasons and pedometer step counts in adulthood (*r* = 0.24–0.30, *p* < 0.001 for total and aerobic steps, which are defined as those taken in bouts of at least 10 min at a pace of 60 steps per minute or more) [22].

### Correlates of ACS

Similar questions were administered for both generations to assess participants’ age, gender, distance to school, and their parents’ education and income. Living area in the G1 questionnaire included four response alternatives (1 = city center, 2 = suburb, 3 = rural community, 4 = dispersed settlement area), which were recategorized as urban (1 and 2) and rural (3 and 4) areas. For G2 participants, corresponding categories were derived from postal codes and the Finnish Environment Institute’s GIS-based classifications of neighborhood urbanity [23]. The school grade within the Finnish educational system was determined based on the participant’s age. Primary school encompassed those aged 8–12, while the age ranges for lower secondary and upper secondary schools were 13–15 and 16–20 years, respectively. Distance to school was reported in kilometers, rounded to the nearest 100 meters. Parental education was categorized as low (comprehensive school: primary and lower secondary school), middle (upper secondary: high school/vocational school), and high (polytechnic and university levels). Due to the limited number of G2 participants with parents having only comprehensive school education, the two lowest categories were combined for further analyses. Parental income was assessed with a seven-class question loosely following the value of money over time and further divided into tertiles: low-, middle-, and high income. In 1983, parental PA was elicited with a three-class question regarding the amount of leisure-time PA. In 2018, the question included six response alternatives, which were recategorized into three to match the 1983 scale. The classes were identified as “low,” “medium,” and “high.” All parental information was collected from both mothers and fathers, and higher values were utilized if information on both parents was available.

### Statistical analyses

The intergenerational differences in the study variables were analyzed with the chi-squared test for the categorical variables and the independent samples *t*-test for the continuous variables.

**Figure 1.**
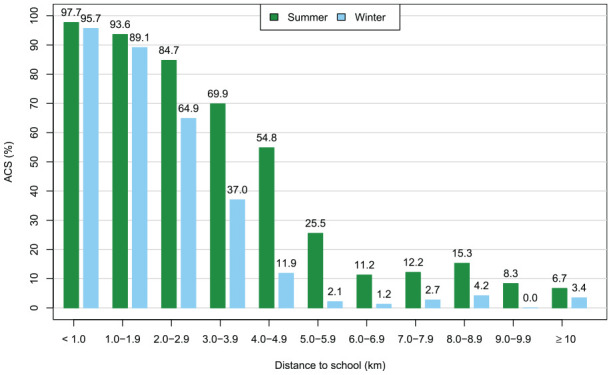
Unadjusted proportions of active commuters during summer and winter seasons according to the distance to school.

To enable a well-adjusted estimation of associations of ACS and other correlates, the potentially dominant effect of commuting distance was first analyzed separately within preliminary models. To this end, the distance was categorized into 1-km blocks. The optimal number of categories was defined by fitting generalized linear models with ACS as the binomial outcome and the commuting distance, categorized into *K* (*K* = 1, . . ., 11) classes, as the only independent variable. The Bayesian information criterion was minimized with *K* = 7 categories. Therefore, for further analysis, the distance was categorized into seven classes: <1, 1–1.9, 2–2.9, 3–3.9, 4–4.9, 5–5.9, and ⩾6 km (Figure 1).

Generalized estimating equation (GEE) models for clustered binary data with logit link functions were utilized to explore the associations of ACS and its correlates, as well as intergenerational differences in these associations. Separate models were constructed for summer and winter commuting. Participants from the same families were indicated by the family ID variable to address the correlation structure of clustered data. A total of 3008 participants with valid data were included in the GEE analyses, comprising parent–child pairs of 455 parents (G1) and their 694 children (G2), as well as 1512 G1 participants and 347 G2 participants without corresponding ACS data from their child or parent (Supplementary Table 1). The correlates of ACS (passive commuting = 0, active commuting = 1) included gender, school grade, distance, living area, parental education, parental income, parental PA, and generation. All correlates were included in a unified GEE framework to simultaneously assess the significance of all associations with the outcome, adjusted for each other.

Potential multicollinearity issues were identified by approximating variance inflation factors (VIFs) for each predictor. Approximate VIFs were obtained by multiple linear regressions between numerically coded predictors, ignoring the multilevel data structure. VIF values higher than 5.0 were interpreted as indicating high multicollinearity. The VIFs ranged from 1.01 (gender) to 1.47 (parental education), indicating no multicollinearity.

The first models included only the main effects of all predictor variables. This approach enabled significance testing of the generation (G1 vs. G2) and other correlates, assuming equal associations of ACS with correlates across generations. Further models included all the main effects and the interaction effects of gender × generation, school grade × generation, living area × generation, parental education × generation, parental income × generation, and parental PA × generation, yielding to significance testing of potential intergenerational transformations of the associations of each correlate. Analyses were performed using R software, version 4.3.1.

### Missing data

In the case of missing data on the correlates of ACS in the original YFS cohort, the 1983 data were supplemented with the baseline information collected in 1980 (or if that was also missing, with the 1986 data), where applicable. The imputed data included parental education (*n* = 49), parental income (*n* = 131), parental PA (*n* = 41), and living area (*n* = 36). For G2 participants with missing values, casewise deletion was applied.

## Results

Commuting modes and correlates of ACS are shown in Table I in the full, combined sample and according to generation. Compared to generation G2, G1 participants were younger, their average distance to school was shorter, and they were more likely to commute actively to school during both seasons (*p* < 0.01). Less than half of the G1 participants lived in urban areas, whereas approximately two-thirds of the G2 participants did. Moreover, the parental educational level was significantly lower in G1 compared to G2 (*p* < 0.001). Significant generational differences were also observed in the distributions of parental income and PA (*p* < 0.001).

**Table I.** Commuting modes and correlates of active commuting to school in the full sample and according to generation.

	All (*N* = 3212)	G1 generation (*n* = 2075)	G2 generation (*n* = 1137)	*p*-value
Age in years, mean (SD)	13.4 (3.3)	13.2 (3.2)	13.6 (3.3)	0.002
School grade				<0.001
Primary (8–12-year-olds)	1539 (47.9)	1098 (52.9)	441 (38.8)	
Lower secondary (13–15-year-olds)	901 (28.1)	550 (26.5)	351 (30.9)	
Upper secondary (16–20-year-olds)	772 (24.0)	427 (20.6)	345 (30.3)	
Gender				0.555
Female	1678 (52.2)	1076 (51.9)	602 (52.9)	
Male	1534 (47.8)	999 (48.1)	535 (47.1)	
Commuting mode in summer				<0.001
Passive	1112 (34.6)	634 (30.6)	478 (42.0)	
Active	2099 (65.3)	1440 (69.4)	659 (58.0)	
Commuting mode in winter				<0.001
Passive	1454 (45.3)	848 (41.0)	606 (53.4)	
Active	1748 (54.4)	1219 (59.0)	529 (46.6)	
Distance to school in km, mean (SD)	5.3 (8.5)	4.8 (7.7)	6.3 (9.7)	<0.001
Distance categories, km				<0.001
<1	798 (24.8)	586 (29.5)	212 (18.9)	
1–1.9	637 (19.8)	427 (21.5)	210 (18.7)	
2–2.9	378 (11.8)	229 (11.5)	149 (13.3)	
3–3.9	229 (7.1)	120 (6.0)	109 (9.7)	
4–4.9	141 (4.4)	86 (4.3)	55 (4.9)	
5–5.9	149 (4.6)	88 (4.4)	61 (5.4)	
⩾6	780 (24.3)	452 (22.7)	328 (29.2)	
Living area				<0.001
Rural	1474 (45.9)	1111 (53.5)	363 (32.3)	
Urban	1725 (53.7)	964 (46.5)	761 (67.7)	
Parental education				<0.001
Comprehensive	1180 (36.7)	1130 (54.5)	50 (4.6)	
Upper secondary	1173 (36.5)	715 (34.5)	458 (42.1)	
Higher	807 (25.1)	228 (11.0)	579 (53.3)	
Parental income				<0.001
Low	769 (23.9)	549 (26.6)	220 (20.8)	
Middle	1024 (31.9)	603 (29.2)	421 (39.9)	
High	1325 (41.3)	910 (44.1)	415 (39.3)	
Parental physical activity				<0.001
Low	549 (17.1)	376 (18.2)	173 (16.0)	
Medium	1764 (54.9)	1089 (52.6)	675 (62.4)	
High	838 (26.1)	605 (29.2)	233 (21.6)	

*Note*: G1: generation 1 (attending school in 1983); G2: generation 2 (attending school in 2018). Values are frequencies (%) unless otherwise noted.

Table II shows the associations between ACS and its correlates, for the summer and winter seasons separately. During the summer, participants in urban areas were more likely to commute actively than those in rural areas (OR = 1.60, 95% confidence interval (CI) [1.21–2.12]). Living area was not associated with the likelihood of ACS during winter. Participants with high parental PA were more likely to commute actively in both seasons (OR = 1.75, 95% CI [1.18–2.60] for summer and OR = 1.58, 95% CI [1.06–2.36] for winter) compared to those with low parental PA.

**Table II.** Correlates of active commuting to school (ACS) during summer and winter seasons in a combined sample of Finnish children and adolescents across two generations.

	ACS during summer			ACS during winter		
	OR	95% CI	*p*-value	OR	95% CI	*p*-value
Gender						
Female	—	—		—	—	
Male	1.03	0.81–1.31	0.795	1.06	0.8–1.36	0.619
School grade						
Primary	—	—		—	—	
Lower Secondary	0.70	0.52–0.94	**0.017**	1.06	0.80–1.40	0.697
Upper Secondary	0.59	0.42–0.82	**0.002**	0.78	0.55–1.11	0.161
Living area						
Rural	—	—		—	—	
Urban	1.60	1.21–2.12	**<0.001**	1.25	0.95–1.66	0.116
Parental education						
Academic	—	—		—	—	
Non-academic	1.03	0.73, 1.45	0.885	0.91	0.65–1.27	0.578
Parental income						
Low	—	—		—	—	
Middle	1.32	0.96–1.82	0.090	1.18	0.82–1.68	0.376
High	1.14	0.82–1.59	0.444	1.10	0.78–1.57	0.580
Parental PA						
Low	—	—		—	—	
Medium	1.34	0.97–1.85	0.073	1.19	0.84–1.68	0.320
High	1.75	1.18–2.60	**0.006**	1.58	1.06–2.36	**0.025**
Generation						
G1	—	—		—	—	
G2	0.62	0.45–0.85	**0.003**	0.73	0.54–1.00	**0.049**
Distance to school, km						
⩽0.9	—	—		—	—	
1–1.9	0.39	0.22–0.68	**<0.001**	0.38	0.25–0.59	**<0.001**
2–2.9	0.15	0.09–0.26	**<0.001**	0.09	0.06–0.13	**<0.001**
3–3.9	0.07	0.04–0.12	**<0.001**	0.03	0.02–0.05	**<0.001**
4–4.9	0.03	0.02–0.06	**<0.001**	0.01	0.00–0.01	**<0.001**
5–5.9	0.01	0.01–0.02	**<0.001**	0.00	0.00–0.00	**<0.001**
⩾6	0.00	0.00–0.01	**<0.001**	0.00	0.00–0.00	**<0.001**

*Note*: ACS: active commuting to school; OR: odds ratio; CI: confidence interval; PA: physical activity; G1: generation 1 (attending school in 1983); G2: generation 2 (attending school in 2018).

Bold values denote statistical significance at the *p* < 0.05 level.

— denotes the reference group.

A higher school grade, belonging to the G2 generation, and greater distance to school were inversely associated with ACS (Table II). Compared to primary school students, lower secondary students were less likely to commute actively during the summer (OR = 0.70, 95% CI [0.52–0.94]), and this likelihood decreased further for upper secondary students (OR = 0.59, 95% CI [0.42–0.82]). School grade was not associated with the likelihood of ACS during winter.

G2 participants were less likely to commute actively than G1 participants in both seasons (OR = 0.62, 95% CI [0.45–0.85] for summer, and OR=0.73, 95% CI [0.54–1.00] for winter). Moreover, the likelihood of ACS significantly decreased as the distance to school increased during both seasons. Participants living 1–1.9 km away from school were less likely to commute actively (OR = 0.39, 95% CI [0.22–0.68] for summer, and OR = 0.38, 95% CI [0.25–0.59] for winter) compared to those living <1 km away, and this likelihood decreased further for greater distances. Gender, parental education, and parental income were not associated with ACS in either season.

**Figure 2.**
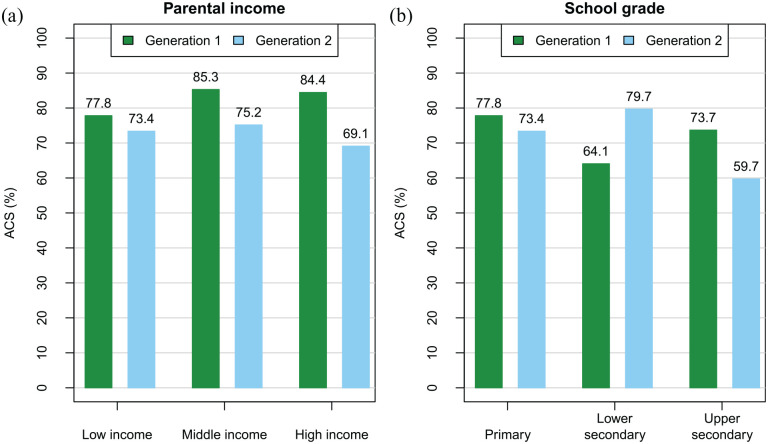
Model estimates on the probability scale for active commuting to school (ACS) during the summer season at distances of 2.0–2.9 km by generation, income class, and school grade: (a) parents’ income classes among G1 and G2, and (b) school grades among G1 and G2. All other correlates have been kept at the reference level.

Generation moderated the associations between lower secondary school grade and ACS (OR = 2.20, 95% CI [1.20–4.05]), as well as between high parental income and ACS (OR = 0.41, 95% CI [0.20–0.84]) during the summer (Table III). To illustrate these associations, we calculated the adjusted probabilities for ACS during summer for different school grades and income classes within the generations, using the interaction model. With the distance to school set at 2.0–2.9 km, the adjusted probability of ACS for lower secondary school students was 80% among G2 and 64% among G1. For the G1 primary school students, it was 78% (Figure 2). Conversely, for students with high parental income, the probability of ACS was higher for G1 (84%) than G2 (69%). No significant interactions with generation were observed for the correlates of winter commuting (Table III).

**Table III.** Intergenerational differences in the correlates of active commuting to school (ACS) during summer and winter seasons.

	ACS during summer			ACS during winter		
	OR	95% CI	*p*-value	OR	95% CI	*p*-value
Gender * Generation						
Male * G2	1.63	0.96–2.77	0.068	0.88	0.53–1.46	0.628
School grade * Generation						
Lower Secondary * G2	2.20	1.20–4.05	**0.011**	1.14	0.64–2.03	0.649
Upper Secondary * G2	0.53	0.28–1.01	0.054	0.56	0.28–1.12	0.100
Living area * Generation						
Urban * G2	1.32	0.74–2.35	0.349	1.31	0.71–2.39	0.386
Parental education * Generation						
Non-academic * G2	0.80	0.39–1.65	0.547	0.69	0.35–1.34	0.272
Parental income * Generation						
Middle * G2	0.52	0.26–1.03	0.060	0.60	0.28–1.26	0.174
High * G2	0.41	0.20–0.84	**0.015**	0.56	0.26–1.18	0.128
Parental PA * Generation						
Some * G2	1.09	0.55–2.16	0.806	0.91	0.44–1.86	0.788
Regular * G2	1.02	0.43–2.39	0.971	1.39	0.59–3.27	0.453

*Note*: ACS: active commuting to school; OR: odds ratio; CI: confidence interval; G1: generation 1 (attending school in 1983); G2: generation 2 (attending school in 2018); PA: physical activity. Models include the interaction effects with generation as well as the main effects of gender (female = ref.), school grade (primary school = ref.), living area (rural = ref.), parental education (academic = ref.), parental income (low income = ref.), parental PA (low PA = ref.), generation (G1 = ref.), and distance to school (⩽0.9 km (ref.), 1–1.9, 2–2.9, 3–3.9, 4–4.9, 5–5.9, and ⩾6 km).

Bold values denote statistical significance at the p < 0.05 level.

## Discussion

In this two-generation study among Finnish children and adolescents, we observed that urban living area and high parental PA were positively associated with ACS. Conversely, a greater distance to school and belonging to the younger generation, that is, attending school in the late 2010s (compared to the early 1980s) were inversely associated with ACS. The associations held for both seasons, except for living area, which was significant only during the summer. Generation moderated the associations of school grade and parental income with ACS during summer: in the 1980s, lower secondary school students were less likely to commute actively than primary school students, a difference not observed in the 2010s. High parental income was associated with a higher likelihood of ACS in the 1980s, but not in the 2010s. Gender and parental education were not associated with ACS.

As expected, distance to school was a key correlate of ACS in both seasons, with the likelihood of ACS decreasing as the distance increased. Consistent with previous research [12], the proportion of active commuters was lower in winter than in summer at all distances, and it declined more steeply at shorter distances in winter. This most likely reflects less frequent cycling during winter [12].

Attending school in the late 2010s, compared to the early 1980s, was associated with a lower likelihood of ACS in both seasons. This aligns with prior research showing a decreasing trend in ACS in various countries, including Finland [13,14], over recent decades. We also observed that urban living area was associated with a higher likelihood of summer ACS compared to rural living areas. This pattern has also been identified in past studies [24–26], and explained by better infrastructure and shorter home–school distances in urban areas. In Finland, a more pronounced decline in ACS from the 1990s to the 2010s has been noted in rural areas compared to urban areas [14], most likely due to increased home–school distances resulting from merging schools into larger units and the closing of smaller rural schools. Over the 35-year span covered by our study, the school network in Finland has been reduced by less than half [27,28]. Concurrently, the proportion of people living in rural areas has decreased, a trend also evident in our data. However, the association between living area and ACS did not vary between generations.

Consistent with previous results indicating the highest probability for ACS at around the end of primary school [15,17], we observed that both lower and upper secondary school grades were associated with a lower likelihood of ACS compared to primary school during summer. However, these effects were moderated by generation. Specifically, lower secondary school was associated with a lower likelihood of ACS compared to primary school only in the 1980s, while for upper secondary school, a trend toward a lower likelihood of ACS was found only in the 2010s. It appears that among the younger generation, the proportion of ACS does not drop until upper secondary school, probably due to factors such as the promotion of PA and active commuting in Finnish society, especially in basic education for Grades 1–9 [29]. Furthermore, the better maintenance of bikeways and improved traffic safety compared to the 1980s have increased the possibility of cycling [30]. However, different age distributions among the two generations obscure the interpretation of these differences. This yields, for example, differences in their access to driving licenses and independent commuting by motorized vehicles.

Generation also moderated the effect of parental income on ACS. High parental income was associated with a higher likelihood of ACS in the 1980s, but not in the 2010s. Conversely, gender and parental education were not associated with ACS. In many high-income countries where ACS prevalence is lower, ACS rates are typically higher among males and those with lower parental education and income [17,19,31]. The associations of education and income have been attributed to shorter home–school distances or less frequent car ownership among those with lower socioeconomic status. The gender difference has been linked to factors such as greater parental permission and fewer safety concerns. However, in Nordic countries with higher levels of ACS and fewer safety concerns, gender has not been a significant factor in ACS [12,32].

Prior research has underscored the role of the family in fostering children’s active behaviors. Both parental commuting behaviors [33] and maternal PA have been linked to higher ACS levels among children [34,35], albeit to a lesser extent compared to parental sociodemographic factors such as education or income [35]. In our study, high parental PA—the only significant predictor of winter commuting besides distance to school and generation—was associated with a higher likelihood of ACS during both seasons. The association between parental PA and children’s ACS could stem from parents’ PA levels reflecting their commuting patterns and general attitudes toward PA and active commuting. Parents are often the primary decision-makers on their children’s commuting modes [36], and their choices are partly explained by their attitudes toward ACS [37].

This study benefited from a unique dataset encompassing children and adolescents from the same families across two generations, separated by 35 years. Similar questions were applied for both generations, which facilitated the exploration of the correlates of ACS and potential intergenerational differences. Commuting habits were assessed separately for summer and winter conditions, allowing for an examination of potential seasonal differences in ACS and its correlates. Several key correlates of ACS, including distance to school, were evaluated. The analyses accounted for the dominant effect of distance through a well-optimized categorization, enabling a well-adjusted estimation of associations between ACS and other correlates.

This study has its limitations. The original cohort, while relatively large and randomly selected, stands in contrast to the more moderately sized and more restricted sample of their children. However, the correlation of observations within the same family was considered in the GEE analyses. The partly dissimilar age distributions of the two samples further limited their comparability. We chose not to distinguish between different modes of active and passive commuting in our analyses. Combining the modes provided more cases for the models, yielding more reliable results on the generational differences in correlates of ACS across different seasons. However, it should be noted that the correlates of walking and cycling could differ. Commuting could also be partially active, such as a combination of public transportation and walking, a factor not considered in this study. Furthermore, different methods were used to classify the living area as urban or rural for G1 and G2. The association of parental education with ACS should also be interpreted with caution, given the imbalanced proportions of higher education between the generations. Lastly, self-reported data on ACS and its correlates introduce potential recall and social desirability biases.

## Conclusions

This study explored ACS and its associated factors among children and adolescents in Finland, spanning two generations attending school in the 1980s and the 2010s. Similar associations were found for both summer and winter conditions, except that fewer statistically significant correlates were identified for winter commuting, which showed less activity. ACS in the present study appears to be equitable regarding gender, parental education, and, as of the 2010s, income as well. A decreasing trend in ACS was noted over time. Support for ACS is particularly necessary in rural areas, and maintaining reasonable distances to school remains a critical priority. The promotion of parental PA should also be considered as a potential strategy for fostering PA and ACS among their children.

## Supplemental Material

sj-docx-1-sjp-10.1177_14034948241304246 – Supplemental material for Correlates of active commuting to school across two generations: the Cardiovascular Risk in Young Finns Study Supplemental material, sj-docx-1-sjp-10.1177_14034948241304246 for Correlates of active commuting to school across two generations: the Cardiovascular Risk in Young Finns Study by Th Suominen, T Kukko, X Yang, K Pahkala, S Rovio, M Hirvensalo, M Kähönen, O Raitakari, Th Tammelin and K Salin in Scandinavian Journal of Public Health
