# Supplementary material for: Correlates of active commuting to school across two generations: the Cardiovascular Risk in Young Finns Study
Source: Scand J Public Health. 2024 Dec 10;53(8):863–71. doi: 10.1177/14034948241304246 (PMC12619840; doi:10.1177/14034948241304246)
Supplement: sj-docx-1-sjp-10.1177_14034948241304246 – Supplemental material for Correlates of active commuting to school across two generations: the Cardiovascular Risk in Young Finns Study [file sj-docx-1-sjp-10.1177_14034948241304246.docx]

**Supplementary Table 1**. Data structure of the G1 and G2 samples showing the family clustering of participants with valid data (n=3008). Values are the numbers of family clusters, with values in brackets representing the numbers of G2 participants

| Number of G2 participants (children) in a cluster | Number of G1 participants (parents) in a cluster | |
| --- | --- | --- |
|  | 0 | 1 |
|  | Number of family clusters [number of G2 participants] | |
| 0 |  | 1512^a^ |
| 1 | 126 [126] | 261 [261] |
| 2 | 78 [156] | 155 [310] |
| 3 | 15 [45] | 36 [108] |
| 4 | 2 [8] | 1 [4] |
| 5 | 0 [0] | 1 [5] |
| 6 | 2 [12] | 1 [6] |
|  | 223 [347^b^] | 455^c^ [694^d^] |

^a^ Number of G1 participants (parents) without corresponding data from their children (G2).

^b^ Number of G2 participants (children) without corresponding data from their parent (G1).

^c^ Number of parents within parent-child pairs

^d^ Number of children within parent-child pairs
